# IGF-1C domain-modified hydrogel enhances therapeutic potential of mesenchymal stem cells for hindlimb ischemia

**DOI:** 10.1186/s13287-019-1230-0

**Published:** 2019-04-29

**Authors:** Nianhuan Zhao, Zhiwei Yue, Jian Cui, Yong Yao, Xianghe Song, Bangping Cui, Xin Qi, Zhibo Han, Zhong-Chao Han, Zhikun Guo, Zuo-Xiang He, Zongjin Li

**Affiliations:** 1 Department of Nuclear Medicine, The First College of Clinical Medical Science, China Three Gorges University, Yichang, 443003 China; 2 Nankai University School of Medicine, 94 Weijin Road, Tianjin, 300071 China; 3 The Key Laboratory of Bioactive Materials, Ministry of Education, The College of Life Science, Nankai University, Tianjin, 300071 China; 4 Department of Intensive Care Unit (ICU), People’s Hospital of Rizhao, Rizhao, 276826 Shandong China; 5 Beijing Tsinghua Changgung Hospital, Tsinghua University, Beijing, 102218 China; 6 Department of Cardiology, Rizhao Hospital of Traditional Chinese Medicine, Rizhao, 276800 Shandong China; 7 Department of Cardiology, Tianjin Union Medical Center, Nankai University Affiliated Hospital, Tianjin, 300121 China; 8 Jiangxi Engineering Research Center for Stem Cell, Shangrao, 334001 Jiangxi China; 9 Henan Key Laboratory of Medical Tissue Regeneration, Xinxiang Medical University, Xinxiang, 453003 China

**Keywords:** Mesenchymal stem cells (MSCs), C domain peptide of insulin-like growth factor-1 (IGF-1C), Hydrogel, Hindlimb ischemia, Angiogenesis, Molecular imaging

## Abstract

**Background:**

Poor cell engraftment and survival after transplantation limited the application of stem cell therapy. Synthetic biomaterials could provide an artificial microenvironment for stem cells, thereby improve cell survival and enhance the therapeutic efficiency of stem cells.

**Methods:**

We synthesized a hydrogel by conjugating C domain peptide of insulin-like growth factor-1 (IGF-1C) onto chitosan (CS-IGF-1C hydrogel). Human placenta-derived mesenchymal stem cells (hP-MSCs), which constitutively express a red fluorescent protein (RFP) and renilla luciferase (Rluc), were co-transplanted with CS-IGF-1C hydrogel into a murine hindlimb ischemia model. Transgenic mice expressing firefly luciferase (Fluc) under the promoter of vascular endothelial growth factor receptor 2 (VEGFR2-Luc) were used. Dual bioluminescence imaging (BLI) was applied for tracking the survival of hP-MSCs by Rluc imaging and the VEGFR2 signal pathway activation by Fluc imaging. To investigate the therapeutic mechanism of CS-IGF-1C hydrogel, angiographic, real-time PCR, and histological analysis were carried out.

**Results:**

CS-IGF-1C hydrogel could improve hP-MSCs survival as well as promote angiogenesis as confirmed by dual BLI. These results were consistent with accelerated skeletal muscle structural and functional recovery. Histology analysis confirmed that CS-IGF-1C hydrogel robustly prevented fibrosis as shown by reduced collagen deposition, along with increased angiogenesis. In addition, the protective effects of CS-IGF-1C hydrogel, such as inhibiting H_2_O_2_-induced apoptosis and reducing inflammatory responses, were proved by in vitro experiments.

**Conclusions:**

Taken together, IGF-1Cs provides a conducive niche for hP-MSCs to exert pro-mitogenic, anti-apoptotic, and pro-angiogenic effects, as well as to inhibit fibrosis. Thus, the incorporation of functional peptide into bioscaffolds represents a safe and feasible approach to augment the therapeutic efficacy of stem cells.

**Electronic supplementary material:**

The online version of this article (10.1186/s13287-019-1230-0) contains supplementary material, which is available to authorized users.

## Background

With considerable morbidity and mortality, the prevalence rate of peripheral arterial disease (PAD) is between 5000 and 10,000 per million people every year [1, 2]. As reported, 4.3~9.6% of patients will end up with amputations due to severe ischemia in their lower extremities [3]. At present, PAD is often treated with minimally invasive surgical interventions such as percutaneous transluminal angioplasty, stent implantation, and artery bypass surgery [4]. However, PAD can lead to critical limb ischemia (CLI) and amputation [5], which appeal for more efforts on the better and the more useful treatments [6]. With their proangiogenic potential, mesenchymal stem cells (MSCs) have been proved for treatment of PAD through enhancing angiogenesis [7–9].

Transplantation of MSCs has been shown to improve perfusion and function in ischemic tissues and reduce amputation rates in models with CLI [10–12]. Unfortunately, the cell survival rate after MSCs transplanted into the ischemic region is quite low, which has limited the application of MSCs [9, 13–15]. Therefore, improving the retention rate after transplantation is one of the critical issues for stem cell-based therapy in limb ischemic disease. The strategy of co-transplantation MSCs with synthetic biomaterials to mimic the microenvironments in vivo has been progressively successful in controlling the transplanted cell fate by imitating the native stem cell niche [13, 16, 17]. Advancing technologies enable novel biomaterials to be designed for promoting cell survival, engraftment into ischemic tissues for persistent function, which will overcome the limited retention of stem cells after delivery [18].

To track the cell fate in vivo after stem cell engraftment is another critical need in the research field. It is essential to understand the behavioral and functional outcome of stem cells within intact living subjects [19, 20]. With the advancement of technology in imaging approaches, one way to realize this is to combine the use of reporter genes, because the signal will not be lost when the cells divide since the reporter gene is replicated with the rest of the genome [21]. The intensity of the signal or the degree of light generated correlates well with the number of cells, so semi-quantitative cell count is possibly achieved by in vivo molecular imaging [19, 21]. As it provides long-term and in vivo visible insights into stem cell transplantation and enables evaluation of the success of stem cell engraftment, molecular imaging offers integral and comprehensive guidance ranging from the basic research to clinical translation of stem cell therapy [22].

In this study, we hypothesized that the novel artificial synthetic bioactive chitosan (CS)-based injectable hydrogel with immobilized C domain peptide of IGF-1 (CS-IGF-1C) [13] could provide a desirable microenvironment for human placenta-derived MSCs (hP-MSCs) to support cell growth and survival and further promote the proangiogenic potential activity of hP-MSCs. To test this hypothesis, we introduced dual reporter gene red fluorescent protein (RFP) and renilla luciferase (Rluc) for the imaging of the survival of hP-MSCs in vivo. We developed a murine model of hindlimb ischemia in transgenic VEGFR2-Luc mice, which expressed firefly luciferase (Fluc) under the promoter of vascular endothelial growth factor receptor 2 (VEGFR2-Luc). Finally, dual bioluminescence imaging (BLI) was applied to monitor the VEGF expression by Fluc imaging and the survival of hP-MSCs by Rluc imaging respectively.

## Materials and methods

### Hydrogel preparation

IGF-1C domain-conjugated chitosan-based hydrogel was synthesized as previously described [13]. In brief, full peptide sequence of IGF-1C domain terminated by the azide group (IGF-1C-N3, N3-GYGSSSRRAPQT) at the N-terminus was synthesized using manual solid phase Fmoc-amino acid chemistry. By condensation chemistry, 6-azido hexanoic acid (Alfa Aesar, Ward Hill, MA) was linked to IGF-1C at the N-terminus to obtain IGF-1C-N3 (N3-GYGSSSRRAPQT). At the same time, with 4-pentynoic acid (Sigma-Aldrich, St. Louis, MO) alkynyl-substituted chitosan (alkynyl-CS) was synthesized by means of the condensation of CS (*M* = 200,000; Jinke, China). Through the click reaction between IGF-1C-N3 and alkynyl-CS, IGF-1C was grafted onto CS. After dialyzed and lyophilized, we get the CS-IGF-1C hydrogel. In order to get gelation, the β-glycerophosphate (β-GP) solution (2.29 M) was added into the CS-IGF-1C solution (3 *w*/*v* %) in an ice bath (about 4 °C). The mixed solution will change to gelation at 37 °C.

### Cell culture

Human placenta-derived MSCs (hP-MSCs) were isolated as previously described [23]. In brief, placentas were donated by pregnant women with prior formal consents. hP-MSCs were collected from chorionic villi. The whole tissue was cut into 1–2-mm pieces and then incubated in 0.2 mg/ml collagenase IV for 90 min. Digested tissue was squeezed through a 100-μm cell strainer to get rid of cell aggregates. Sieved cell suspension was washed twice with PBS and re-suspended in culture medium of Dulbecco minimum essential medium (DMEM)/F12 (Gibco, Grand Island, NY), containing 10% fetal bovine serum (FBS, HyClone, Australia) and supplemented with 1% 100 U/ml penicillin/streptomycin (Gibco) and 1% non-essential amino acid (NEAA) [24]. For in vitro study, the experiment was divided into three groups: control group (cells cultured with medium only), CS group (cells cultured with CS), and CS-IGF-1C group (cells cultured with CS-IGF-1C hydrogel). Furthermore, the morphology of hP-MSCs seeded into CS or IGF-1C hydrogel after lyophilization was examined using scanning electron microscopy (SEM; Phenom, Shanghai, China). To track cells in vivo, hP-MSCs were transduced at a multiplicity of infection of 10 with a self-inactivating lentiviral vector carrying an EF1α promoter driving renilla luciferase (Rluc) and red fluorescent protein (RFP) double-fusion (DF) reporter genes [25].

### Phenotype features of hP-MSCs

The phenotype of hP-MSCs-Renilla-RFP was detected by flow cytometry. The hP-MSCs were dissociated with 0.25% trypsin-EDTA (Gibco, Grand Island, NY) and then washed with PBS containing 2% FBS. Afterwards, the cell suspensions were incubated with fluorescence-conjugated antibodies including CD44, CD90, CD31, and CD45 (BD Biosciences, San Jose, CA) for 30 min. The FACS analysis was performed using a FACS CaliburTM flow cytometer (BD Biosciences), and the data were analyzed using the Cell Quest Pro software (BD Biosciences).

### Cells proliferation assay

For proliferation test, hP-MSCs were seeded in 12-well plates coated with CS, or CS-IGF-1C hydrogel or uncoated plates at a density of 100,000 cells/well. At a different time point (24 h, 48 h, and 72 h), bioluminescence imaging (BLI) was performed to evaluate cell survival as described previously [26].

### Quantitative determination of antioxidative stress

In order to investigate the anti-oxidative stress effects of CS-IGF-1C hydrogel, 6-well plates were coated with CS or CS-IGF-1C hydrogel. A total of 5 × 10^5^ hP-MSCs per well were seeded and subcultured for 12 h. Then, we changed the medium to complete medium and treated with hydrogen peroxide (H_2_O_2_, 500 μM) for 4 h. And cell survival was determined by BLI. To determine the pathway involved in this protective action, we also measured the expression of apoptosis-related genes in hP-MSCs after treatment with real-time PCR.

### Cell transplantation

The therapeutic contribution of MSCs to ischemic diseases includes direct differentiation into the endothelial cells or by promoting angiogenesis through paracrine effects [7]. To investigate whether IGF-1C hydrogel could improve the angiogenic activity of MSCs, transgenic mice expressing firefly luciferase (Fluc) under the promoter of vascular endothelial growth factor receptor 2 (VEGFR2-Luc) were used [9, 24]. Mice were raised under a specific pathogen free (SPF) animal area at the Animal Facility of Nankai University. The protocols about the treatment of animals and the experimental procedures of the present study were approved by the Nankai University Animal Care and Use Committee guidelines that conform to the Guidelines for Animal Care approved by the National Institutes of Health (8th Edition, 2011). The mouse hindlimb ischemia (HLI) model was set up as previously described [9, 24]. After surgery, 1 × 10^6^ hP-MSCs stably expressing Rluc-RFP cells were injected into unit 4 quadriceps and gastrocnemius muscles at three different points post-surgery at 30 μl total volume suspended in PBS, CS, or CS-IGF-1C hydrogel, respectively. The control mice were injected with an equal volume of PBS (ten mice in each group).

### Bioluminescence imaging

To investigate the fate and the treatment effect of the transplanted cells in vivo, hP-MSCs labeled with Rluc and RFP were used. After cell transplantation, BLI analysis was performed longitudinally to track the survival of hP-MSCs in the HLI model. At the indicated time points (days 0, 2, 4, 6, and 8), water-soluble coelenterazine (5 mg/kg; Nanolight Technology, Pinetop, AZ), a substrate of Rluc, was injected via the tail vein. Animals were imaged immediately using the IVIS Lumina Imaging System (Xenogen Corporation, Hopkinton, MA). To track the VEGFR2 expression following hP-MSCs transplantation, mice were intraperitoneally injected with d-luciferin (150 mg luciferin/kg). BLI was conducted on days 3, 7, 10, 14, and 21.

### Assessment of limb perfusion and function

To assess the limb perfusion and angiogenesis, angiography was performed to evaluate the vessel density in ischemic limbs (*n* = 5 for each group) on day 21. The contrast agent barium sulfate (0.3 g/ml, 10 ml) was infused into the blood vessels. Images were acquired by using the Kodak In-Vivo FX Pro Imaging System (Kodak, New Haven, CT) for quantitative angiographic analysis [24]. Vessel density was measured using ImageJ software. A semi-quantitative functional assessment of the ischemic limbs was performed by a blinded observer using a modification of a clinical score system as described before [24].

### Histological analysis

For angiogenesis analysis, animals were euthanized and the muscle tissues were embedded into OCT compound (Sakura Finetek, Japan). Samples were cut into 6-μm-thick sections for immunofluorescence staining. The sections were incubated with primary antibody against CD31 (rat anti-mouse, Abcam, Cambridge, MA) overnight at 4 °C, then incubated with Alexa Fluor 594 goat anti-rat IgG (Invitrogen, Grand Island, NY) and counterstained with 6-diamidino-2-phenylindole (DAPI; Southern Biotech, Birmingham, AL). The number of capillary vessels was randomly counted in ten selected areas with a fluorescence microscope (× 200). Hematoxylin-eosin (HE) staining and Masson’s trichrome staining were performed to investigate the therapeutic effects of IGF-1C hydrogel and hP-MSCs on day 21.

### Quantitative RT-PCR

Total RNA was extracted from cells or muscle samples with 500 μl TRIzol (Invitrogen, Grand Island, NY) following the manufacturer’s protocol. Subsequently, the First-strand cDNA Synthesis System (TransGen Biotech, Beijing, China) was used to reversely transcribe total RNA into cDNA. Real-time PCR (Bio-Rad, Hercules, CA) using FastStart Universal SYBR Green Master (Roche, Mannheim, Germany) was applied to quantify the expressions of relative genes expression. Then, relative gene expression fold changes were identified with the 2^−ΔΔCt^ method. The sequences of primers used in this study were shown in Additional file 1: Tables S1 and S2.

### Statistical analyses

All data are expressed as standard error of the mean (SEM). One-way analysis of variance was used. Differences were considered significant at *P* values < 0.05. All results presented are from at least three independent experiments for each condition.

## Results

### Labeling of hP-MSC with reporter genes

**Fig. 1:**
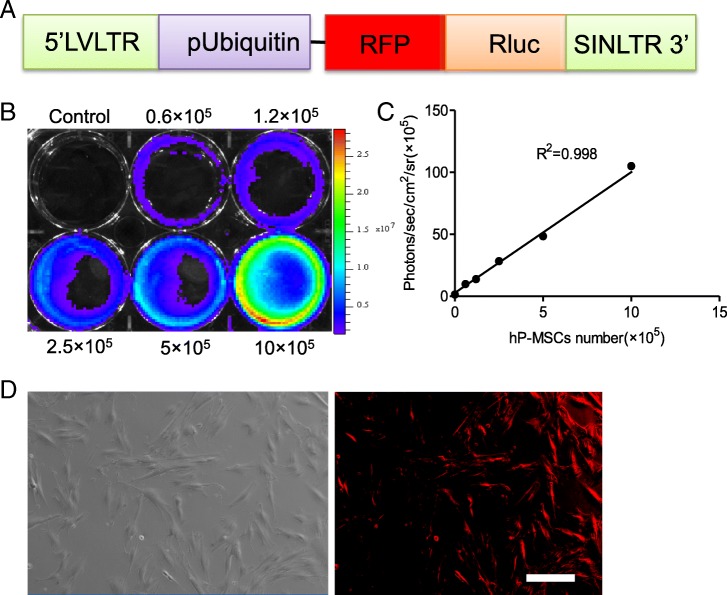
Characterization of the double fusion (DF) renilla luciferase (Rluc) and red fluorescent protein (RFP) hP-MSCs. **a** Schematic of the DF reporter gene containing Rluc and RFP driven by a ubiquitin promoter. **b**, **c** BLI quantification demonstrated a significant linear relationship between the number of hP-MSCs and average radiance of Rluc. **d** hP-MSCs are strongly positive for RFP on fluorescence microscopy. Scale bar, 200 μm

In this study, hP-MSCs were transfected with a double-fusion reporter gene, renilla luciferase (Rluc) and red fluorescent protein (RFP) (Fig. 1a). And BLI exhibited a robust linear correlation between Rluc-labeled hP-MSCs number and optical intensity of Rluc average radiance (*R*^2^ = 0.998, Fig. 1b, c). Immunofluorescence assays revealed that RFP was robustly expressed on hP-MSCs (Fig. 1d). We obtained the CS-based injectable hydrogel grafted with IGF-1C (CS-IGF-1C) (Additional file 1: Figure S1A). And the C domain of IGF-1 is a 12 amino acid sequence of GYGSSSRRAPQT [27]. The CS-IGF-1C hydrogel used in this study has an IGF-1C concentration 2.0 mg/ml [2.0‰ (*w*/*v*)], which showed excellent biocompatibility and bioactivity in previous study [13]. The proliferation of hP-MSCs was better on CS-IGF-1C hydrogel than on CS (Additional file 1: Figure S1B). Moreover, flow cytometry analysis results revealed that the phenotype of hP-MSCs was barely influenced after reporter gene insertion (Additional file 1: Figure S1C).

### Protective effects of CS-IGF-1C hydrogel in vitro

**Fig. 2:**
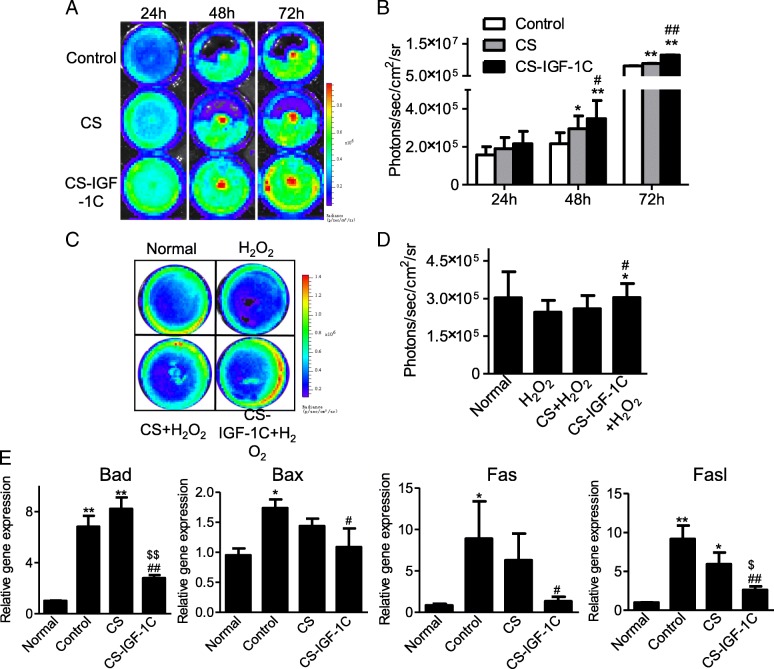
Biocompatibility of CS-IGF-1C hydrogel. **a** BLI revealed that CS-IGF-1C hydrogel enhanced the proliferation of hP-MSCs. **b** Quantitative analysis of BLI signals. The signal activity expressed as photons/sec/cm^2^/steradian. *P < 0.05, **P < 0.01 versus control; ^#^*P* < 0.05, ^##^*P* < 0.01 versus CS. **c** Anti-apoptotic effects of CS-IGF-1C hydrogel. BLI analysis data revealed that CS-IGF-1C hydrogel promoted hP-MSCs survival upon the exposure to H_2_O_2_ (500 μM). hP-MSCs cultured with medium only, CS, or CS-IGF-1C hydrogel, and then exposed to H_2_O_2_ for 4 h. **d** Quantification of BLI signals displayed that CS-IGF-1C hydrogel could rescue the oxidative stress of hP-MSCs after H_2_O_2_ treatment. **e** Real-time quantitative PCR test showed the expression of apoptosis-related genes expression of hP-MSCs after treated with H_2_O_2_ (500 μM) for 4 h. All data were shown as means ± SEM in triplicate assays. *P < 0.05, **P < 0.01 vs normal; ^#^*P* < 0.05, ^##^*P* < 0.01 vs H_2_O_2_; ^$^*P* < 0.05, ^$$^*P* < 0.01 vs H_2_O_2_ + CS

In this study, BLI was employed to measure cell proliferation at 24 h, 48 h, and 72 h after treated with CS or CS-IGF-1C hydrogel (Fig. 2a). As shown in Fig. 2b, all results suggested that the cell proliferation activity was increased by CS-IGF-1C hydrogel compared with the control group and CS group. Then, we investigated whether the CS-IGF-1C hydrogel supports the viability of hP-MSCs in culture under oxidative condition. In particular, the oxidative stress will trigger apoptosis during ischemia injury, further exacerbates initial tissue damages. In the present study, hP-MSCs were treated with H_2_O_2_, then BLI was used to determine whether CS-IGF-1C hydrogel can support the survival of hP-MSCs under oxidative stress, which is a major cause of low cell survival in ischemic tissues after transplantation [17]. The survival of hP-MSCs was judged by bioluminescence intensity. The BLI revealed robust signals in the CS-IGF-1C hydrogel group (Fig. 2c, d). H_2_O_2_ treatment increased apoptosis genes Bad, Bax, Fas, and FasL expression in hP-MSCs. Cultured with CS remarkably reduced the expression of those apoptosis genes, which was further decreased by CS-IGF-1C hydrogel treatment (Fig. 2e). These results indicated that CS-IGF-1C hydrogel could protect hP-MSCs against oxidative stress.

### Improvement of cell survival in vivo

**Fig. 3:**
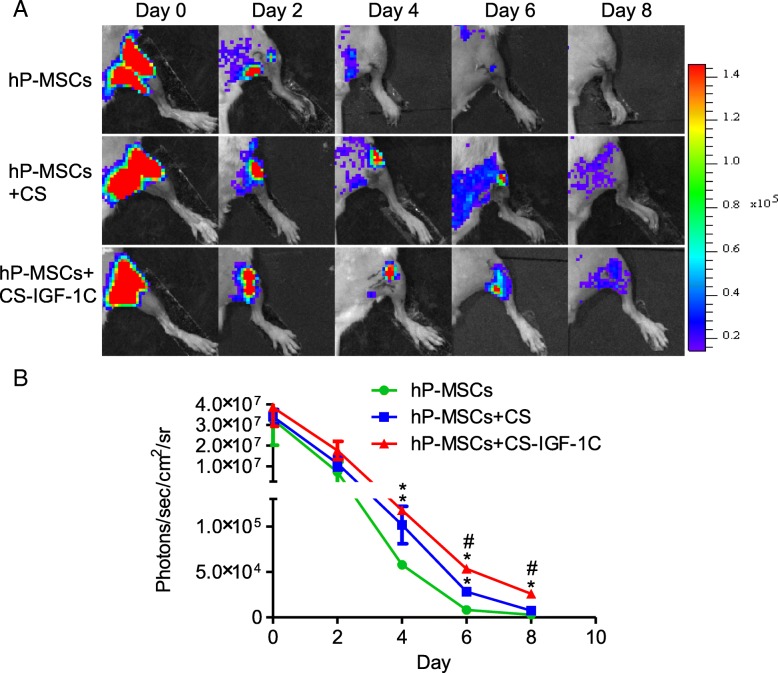
CS-IGF-1C hydrogel increased hP-MSCs retention in vivo. **a** The fate of hP-MSCs after transplantation was tracked by BLI. Mice received 1 × 10^6^ hP-MSCs alone, hP-MSCs with CS, or hP-MSCs with CS-IGF-1C hydrogel. **b** Quantitative analysis of BLI signals demonstrated that cell survival was improved by the application of CS-IGF-1C hydrogel. Data are expressed as mean ± SEM. *P < 0.05 versus hP-MSCs; ^#^*P* < 0.05 versus hP-MSCs co-transplanted with CS hydrogel

Scanning electron microscopy (SEM) images demonstrated the distribution of hP-MSCs before transplantation (Additional file 1: Figure S2). After cell transplantation, we performed the BLI analysis to longitudinally track the survival of hP-MSCs in the HLI model. All groups showed similar robust signals by BLI immediately after cell transplantation, which revealed a successful delivery of an equal number of cells. Over time, the signals of bioluminescence decreased in all groups. CS-IGF-1C hydrogel could significantly improve cell survival and further increase the therapeutic potential of hP-MSCs (Fig. 3a, b).

### Enhancement of angiogenesis in HLI model

**Fig. 4:**
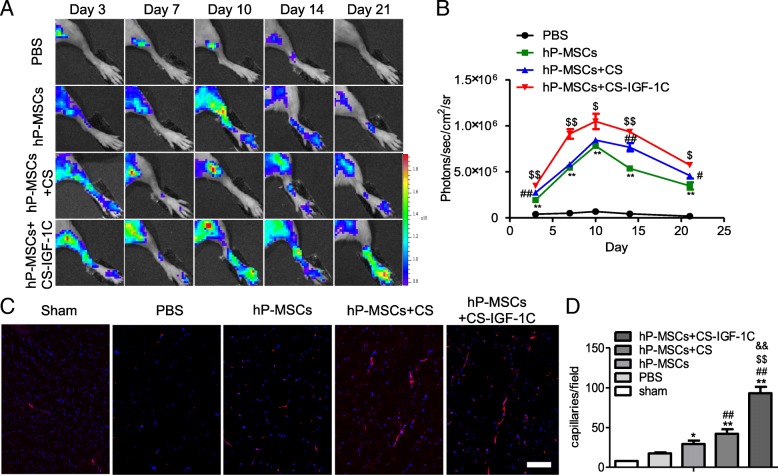
CS-IGF-1C hydrogel augmented the proangiogenic effects of hP-MSCs. **a** BLI tracked the VEGFR2-Fluc expression following hP-MSCs transplanted in HLI models. Colored scale bar represents the bioluminescence average radiance in photons/s/cm^2^/steradian. **b** Quantitative analysis data revealed the improved proangiogenic effects of CS-IGF-1C hydrogel. Data are expressed as mean ± SEM. *P < 0.05 versus PBS; ^#^*P* < 0.05 versus hP-MSCs; ^$^*P* < 0.05 versus hP-MSCs + CS. **c** Representative images of muscle sections stained for CD31 (red) at day 14. Nuclei were counter stained with DAPI. **d** Quantitative analysis of CD31 immunostaining in each group. The number of capillary vessels was counted in five randomly selected areas. Scale bar, 100 μm. Data are expressed as mean ± SEM. *P < 0.05, **P < 0.01 versus sham; ^##^*P* < 0.01 versus PBS; ^$$^*P* < 0.01 versus hP-MSCs; ^&&^*P* < 0.01 versus hP-MSCs + CS hydrogel

**Fig. 5:**
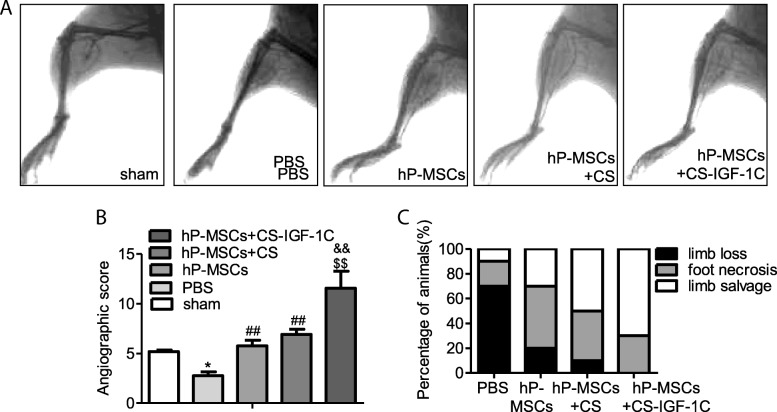
CS-IGF-1C hydrogel co-transplanted with hP-MSCs enhanced blood perfusion of the injured hindlimb. **a** Photograph of angiography on day 21 showed the collateral vessels in the ischemic hindlimb. All mice models were in prone position for photos. **b** Angiography score indicated that treated with hP-MSCs and CS-IGF-1C hydrogel significantly augmented the collateral vessels. Data are expressed as the mean ± SEM. *P < 0.05, **P < 0.01 versus sham; ^##^*P* < 0.01 versus PBS; ^$$^*P* < 0.01 versus hP-MSCs; ^&&^*P <* 0.01 versus hP-MSCs + CS. **c** Percentage distributions of limb salvage, foot necrosis, and limb loss in each group on day 14

BLI technology allows real-time monitoring of the angiogenesis in VEGFR2-Luc mice [19]. VEGFR2 is the main signaling receptor for VEGF and is expressed primarily on endothelial cells. The luciferase signal was imaged for each mouse on days 3, 7, 10, 14, and 21 (Fig. 4a). We observed a peak of luciferase activity on the tenth day in all groups, and the strongest signal was detected in the CS-IGF-1C hydrogel and hP-MSCs co-transplantation group, which suggested that the VEGF/VEGFR2 pathway was activated and CS-IGF-1C hydrogel could increase this angiogenic effect (Fig. 4b). Furthermore, immunostaining of CD31 on day 14 was conducted. HLI mice treated with CS-IGF-1C hydrogel and hP-MSCs had the highest capillary density compared with the control groups on day 14 (Fig. 4c, d). On day 21 post-surgery, mice underwent angiography to investigate the collateral vessel development. Representative photographs are shown in Fig. 5a. All results implied that CS-IGF-1C hydrogel augmented the angiogenic actions of hP-MSCs (Fig. 5b). hP-MSCs with CS-IGF-1C hydrogel significantly ameliorated the generation of collateral vessels at the ischemia sites.

### Improvement of limb function

**Fig. 6:**
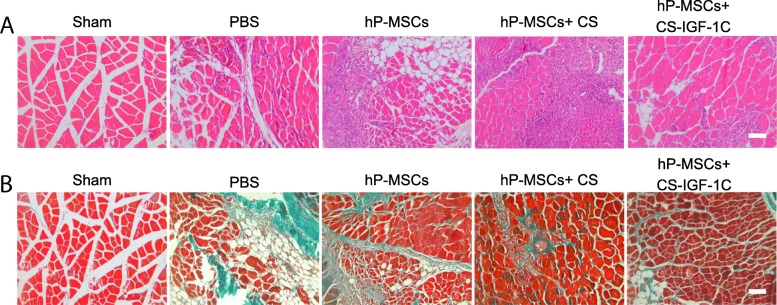
CS-IGF-1C hydrogel co-transplanted with hP-MSCs promoted the recovery of hindlimb function. **a** HE-stained muscle sections on day 28. **b** Masson’s trichrome-stained muscle sections on day 28. Experiments were performed in triplicate. Scale bars, 50 μm

We next investigated the therapeutic potential of hP-MSCs injected with CS-IGF-1C hydrogel. The severity of the injury was evaluated to examine the therapeutic effects of hP-MSCs on day 14. As shown in Fig. 5c, mice treated with hP-MSCs and CS-IGF-1C hydrogel had significantly lower limb loss or foot necrosis and achieved better limb salvage. We concluded that hP-MSCs combined with CS-IGF-1C hydrogel could accelerate ischemia limb function recovery and promote limb regeneration. Furthermore, we performed HE staining of muscle tissue on day 28. HE staining of the ischemic limb muscles revealed extensive muscle protection in the group treated with hP-MSCs and CS-IGF-1C hydrogel (Fig. 6a). We next performed Masson’s staining to evaluate tissue fibrosis in the muscle samples on day 28. Masson’s staining revealed that CS-IGF-1C hydrogel significantly suppressed collagen deposition (Fig. 6b).

### Angiogenic effects of CS-IGF-1C hydrogel

**Fig. 7:**
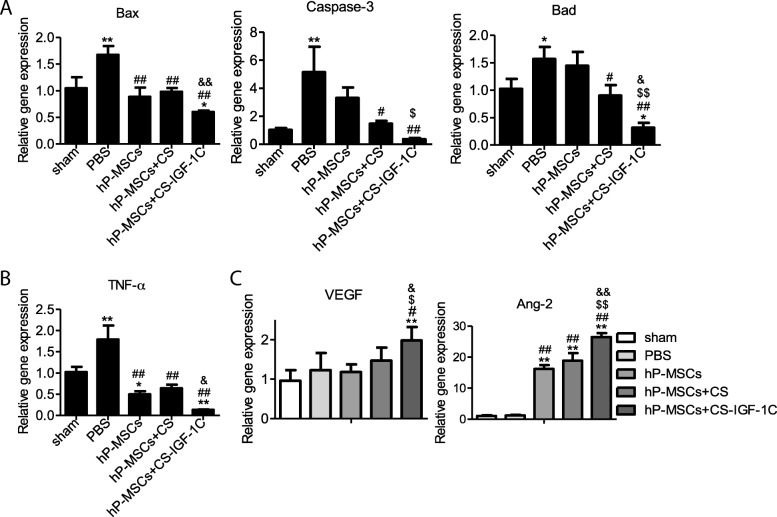
Therapeutic mechanisms of CS-IGF-1C hydrogel for hindlimb ischemia. **a** Apoptotic-related gene expression. The expression levels of Bax, Caspase-3, and Bad were evaluated by RT-PCR in each group on day 14. **b** The expression level of TNF-α was evaluated by RT-PCR in each group on day 14. **c** Expression levels of VEGF and Ang-2 were evaluated by RT-PCR in each group on day 14. Data were expressed as mean ± SEM. *P < 0.05, **P < 0.01 versus sham; ^#^*P* < 0.05, ^##^*P* < 0.01 versus PBS; ^$^*P* < 0.05 versus hP-MSCs; ^&^*P* < 0.05 versus hP-MSCs + CS

To gain insight into the mechanisms of CS-IGF-1C hydrogel-induced angiogenesis, real-time RT-PCR analysis was carried out. The results revealed that apoptosis-related gene Bad, Caspase-3, and Bax and inflammatory-related factor TNF-α were downregulated (Fig. 7a, b), and the angiogenic factors VEGF and Ang-2 were highly upregulated (Fig.7c) in the ischemic limbs of mice injected with hP-MSCs and CS-IGF-1C hydrogel on day 14. All results implied that CS-IGF-1C hydrogel could enhance neovascularization and improve limb salvage.

## Discussion

**Fig. 8:**
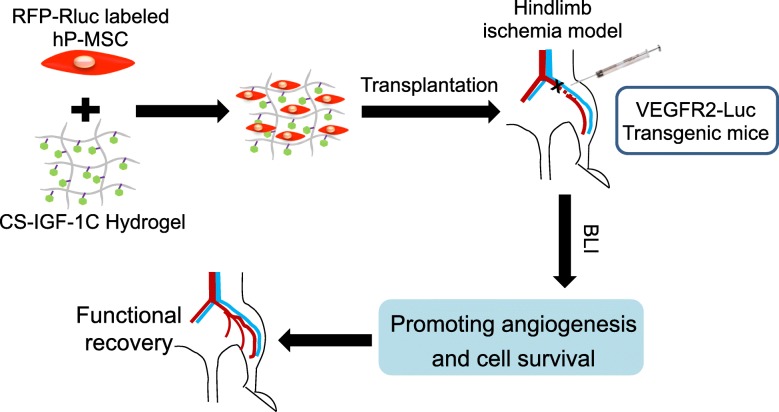
Schematic diagram depicts the therapeutic effects of CS-IGF-1C hydrogel with hP-MSCs for treatments of hindlimb ischemia. In this study, dual BLI was applied for tracking the survival of hP-MSCs by Rluc imaging and the VEGFR2 signal pathway activation by Fluc imaging. When co-transplanted into the hindlimb ischemia model, CS-IGF-1C hydrogel could enhance the survival of hP-MSCs and further promote function recovery of the injured hindlimb by ameliorating angiogenesis and alleviating fibrosis

In this study, we demonstrated a biocompatible scaffold CS-IGF-1C hydrogel could provide a mimic extracellular matrix (ECM) for hP-MSCs after transplanted into the hypoxic region in mice model of HLI. Our results revealed that CS-IGF-1C hydrogel could improve neovascularization and promote the functional recovery of the hindlimb through promoting angiogenesis and inhibiting apoptosis pathway. Moreover, dual BLI is an invaluable tool for tracking cell survival as well as the proangiogenic effects of hP-MSCs (Fig. 8).

With the increasing interests in the use of MSCs for CLI therapy [28], the low retention rate of MSCs after delivery has become one of the serious barriers to clinical application. In this study, CS-IGF-1C hydrogel greatly increased the engraftment and retention of transplanted hP-MSCs in vivo, which could prolong the therapeutic effects of hP-MSCs. A number of biologic materials have been developed to be used in regenerative medicine area, for instance, a type of injectable scaffolds of hydroxyapatite-polymer nanocomposite microspheres [29], a porous bi-layered poly (lactide-co-glycolide) (PLG) scaffold system [30], a polysaccharide-based biomaterial with nitric oxide-releasing property, and so on. For hydrogel, its special features are suitable for the biomedical application, including sustained release, bioactivity, and degradability [31].

Ischemia diseases are often caused by a partial or complete obstruction in the arterial trees leading to acute systemic responses characterized by increased oxidative stress, endothelial damages, and inflammation. Accumulating evidences show that inflammation plays important roles in the initiation and progression of PAD, and the level of inflammatory markers is associated with the severity of PAD [28, 29]. In our investigation, CS-IGF-1C hydrogel had the ability to reduce the inflammatory responses, like downregulating the expression of TNF-α gene, and to inhibit cell apoptosis. In our study, the injectable CS-IGF-1C hydrogel could provide a biomimetic niche to mediate hP-MSCs activity at the molecular level. For instance, CS-IGF-1C hydrogel promoted the expression of genes associated with cell proliferation (such as VEGF) and reduced inflammatory response (such as TNF-α). CS-IGF-1C hydrogel is a kind of thermosensitive hydrogel, which is an ideal injectable scaffold to deliver stem cells [18, 32–34].

Non-invasive tracking by reporter gene has been well established to longitudinally monitor the proliferation, migration, apoptosis, and progress in the pathophysiology of the injected cells [17]. Other than monitoring the survival status of transplanted cells in vivo, molecular imaging modalities also offer the potential for real-time assessment of molecule changes in vivo, which could provide direct evidences for the disclosure of the therapeutic mechanisms of stem cell transplantation [20]. The use of MSCs for therapeutic angiogenesis has been confirmed in animal models and pre-clinical studies, and evidences have revealed that MSCs could promote neovascularization and restore blood flow to ischemic limbs [7]. In this study, dual BLI was utilized for monitoring the survival of hP-MSCs, as well as the activation of VEGF/VEGFR2 pathway. Based on the BLI, obvious improvement of angiogenesis was observed in co-transplantation of hP-MSCs with CS-IGF-1C hydrogel, suggesting that this hydrogel could upregulate the VEGF/VEGFR2 pathway in mice, which finally resulted in the functional recovery of hindlimb ischemia.

Biomaterials are designed to meet the various strategies for regenerative medicine, such as providing a scaffold for cell adhesion and proliferation and serving as carriers of bioactive factors [35]. Immobilization of growth factors by binding to a matrix to mimic stem cell niche provides extra cues for stem cell survival and differentiation. A number of motifs from growth factors have been identified that can mimic functions of full-length proteins [36]. Short peptides are easily synthesized and have lower antigenicity than full-length proteins. In addition, a small number of residues are also advantageous for the improvement of binding affinity to the matrix [37]. Thus, this strategy was employed in our study to determine the biological activity of immobilized IGF-1C peptide in cell-based therapy. An ideal biomaterial designed for stem cell transplantation should be able to create a novel bioactive material close to the natural matrix components. Decellularized extracellular matrix (dECM) scaffold, such as from MSCs, serves as a novel stem cell carrier and is promising for tissue engineering and regenerative medicine [38].

## Conclusion

In summary, IGF-1C immobilized on a chitosan hydrogel could provide an artificial microenvironment for hP-MSCs and thereby improve cell survival in a HLI model. CS-IGF-1C hydrogel can obviously improve the therapeutic efficacy of hP-MSCs for HLI disease by improving the retention rate of transplanted cells and promoting angiogenesis and blood flow recovery. This study offers a new opportunity for the use of CS-IGF-1C hydrogel in the treatment of ischemic diseases.

## Additional files

Additional file 1: **Table S1.** RT-PCR primer sequences (human). **Table S2.** RT-PCR primer sequences (mouse). **Figure S1.** Characterization of CS-IGF-1C hydrogel. (A) The chemical structural formula of CS and CS-IGF-1C. With a reaction between IGF-1C-N3 and alkynyl-CS, IGF-1C was grafted onto CS hydrogel. (B) Morphology of hP-MSCs of CS-IGF-1C hydrogel. Optical images of hP-MSCs cultured with CS or CS-IGF-1C hydrogel after 12 h. Scale bar, 200 μm. (C) Reporter gene insertion barely influences the phenotype of hP-MSCs with flow cytometry. **Figure S2.** Representative SEM images. SEM images of CS-IGF-1C hydrogel with hP-MSCs (left) or not (right). Arrows indicate hP-MSCs. Scale bar, 20 μm. **Figure S3.** Three outcomes to estimate the therapeutic effect. The photograph of three therapeutic outcomes: limb salvage, foot necrosis, and limb loss. (DOCX 1647 kb)

## Abbreviations

3D: Three-dimensional
BLI: Bioluminescence imaging
CLI: Critical limb ischemia
CS: Chitosan
DMEM: Dulbecco minimum essential medium
FBS: Fetal bovine serum
Fluc: Firefly luciferase
HE: Hematoxylin-eosin
HLI: Hindlimb ischemia
hP-MSCs: Human placenta-derived mesenchymal stem cells
IGF-1C: Insulin-like growth factor-1C
MSCs: Mesenchymal stem cells
NEAA: Non-essential amino acid
p/s/cm^2^/sr: Photons/cm^2^/second/steradian
PAD: Peripheral arterial disease
PBS: Phosphate-buffered saline
RFP: Red fluorescent protein
Rluc: Renilla luciferase
ROI: Region of interest
SEM: Standard error of the mean
VEGFR2: Vascular endothelial growth factor receptor 2
β-GP: β-Glycerophosphate
