# Supplementary material for: IGF-1C domain-modified hydrogel enhances therapeutic potential of mesenchymal stem cells for hindlimb ischemia
Source: Stem Cell Res Ther. 2019 Apr 29;10:129. doi: 10.1186/s13287-019-1230-0 (PMC6489284; doi:10.1186/s13287-019-1230-0)
Supplement: Supplementary file 1 — Table S1. RT-PCR primer sequences (human). Table S2. RT-PCR primer sequences (mouse). Figure S1. Characterization of CS-IGF-1C hydrogel. (A) The chemical structural formula of CS and CS-IGF-1C. With a reaction between IGF-1C-N3 and alkynyl-CS, IGF-1C was grafted onto CS hydrogel. (B) Morphology of hP-MSCs of CS-IGF-1C hydrogel. Optical images of hP-MSCs cultured with CS or CS-IGF-1C hydrogel after 12 h. Scale bar, 200 μm. (C) Reporter gene insertion barely influences the phenotype of hP-MSCs with flow cytometry. Figure S2. Representative SEM images. SEM images of CS-IGF-1C hydrogel with hP-MSCs (left) or not (right). Arrows indicate hP-MSCs. Scale bar, 20 μm. Figure S3. Three outcomes to estimate the therapeutic effect. The photograph of three therapeutic outcomes: limb salvage, foot necrosis, and limb loss. (DOCX 1647 kb) [file 13287_2019_1230_MOESM1_ESM.docx]

**IGF-1C Domain Modified Hydrogel Enhances Therapeutic Potential of Mesenchymal Stem Cells for Hindlimb Ischemia**

Nianhuan Zhao, Zhiwei Yue, Jian Cui, Yong Yao, Xianghe Song, Bangping Cui, Xin Qi, Zhibo Han, Zhong-Chao Han, Zhikun Guo, Zuo-Xiang He, Zongjin Li

**Additional file 1**

**Table S1. RT-PCR primer sequences (human).**

| **Gene** | **Forward(5'to 3’)** | **Reverse(5'to 3’)** |
| --- | --- | --- |
| GAPDH | GGAGCGAGATCCCTCCAAAAT | GGCTGTTGTCATACTTCTCATGG |
| Bad | CCCAGAGTTTGAGCCGAGTG | CCCATCCCTTCGTCGTCCT |
| Bax | CCCGAGAGGTCTTTTTCCGAG | CCAGCCCATGATGGTTCTGAT |
| Fas | TCTGGTTCTTACGTCTGTTGC | CTGTGCAGTCCCTAGCTTTCC |
| Fasl | TGCCTTGGTAGGATTGGGC | GCTGGTAGACTCTCGGAGTTC |

**Table S2. RT-PCR primer sequences (mouse).**

| **Gene** | **Forward(5'to 3’)** | **Reverse(5'to 3’)** |
| --- | --- | --- |
| GAPDH | GGAGAGTGTTTCCTCGTCCC | ACTGTGCCGTTGAATTTGCC |
| VEGF | CACGACAGAAGGAGAGCAGAA | ATGTTGCTCTCTGACGTGGG |
| Ang-2 | CAGCCACGGTCAACAACTC | CTTCTTTACGGATAGCAACCGAG |
| TNF-α | GACGTGGAACTGGCAGAAGA | ACTGATGAGAGGGAGGCCAT |
| Bax | AGACAGGGGCCTTTTTGCTAC | AATTCGCCGGAGACACTCG |
| Caspase-3 | GGCACAAAGCGACTGGATG | CTGCCGTGGTACAGAACTGG |
| Bad | TGAGCCGAGTGAGCAGGAA | GCCTCCATGATGACTGTTGGT |

**Supplementary figure**

**
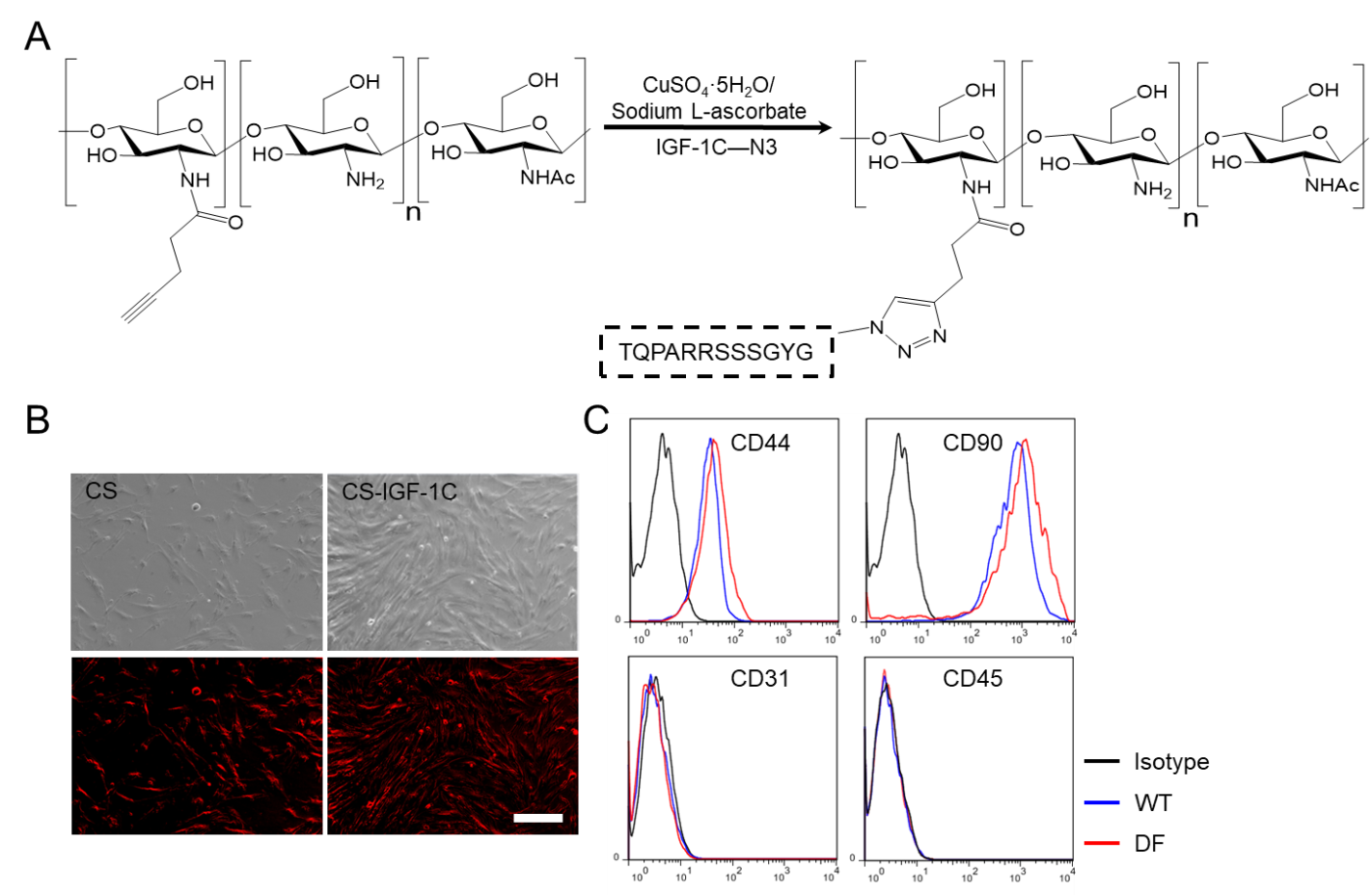
**

**Supplementary Fig. 1: Characterization of CS-IGF-1C hydrogel. (A)** The chemical structural formula of CS and CS-IGF-1C. With a reaction between IGF-1C-N3 and alkynyl-CS, IGF-1C was grafted onto CS hydrogel. **(B)** Morphology of hP-MSCs of CS-IGF-1C hydrogel. Optical images of hP-MSCs cultured with CS or CS-IGF-1C hydrogel after 12h. Scale bar, 200 μm. **(C)** Reporter genes insertion barely influences the phenotype of hP-MSCs with flow cytometry.

**
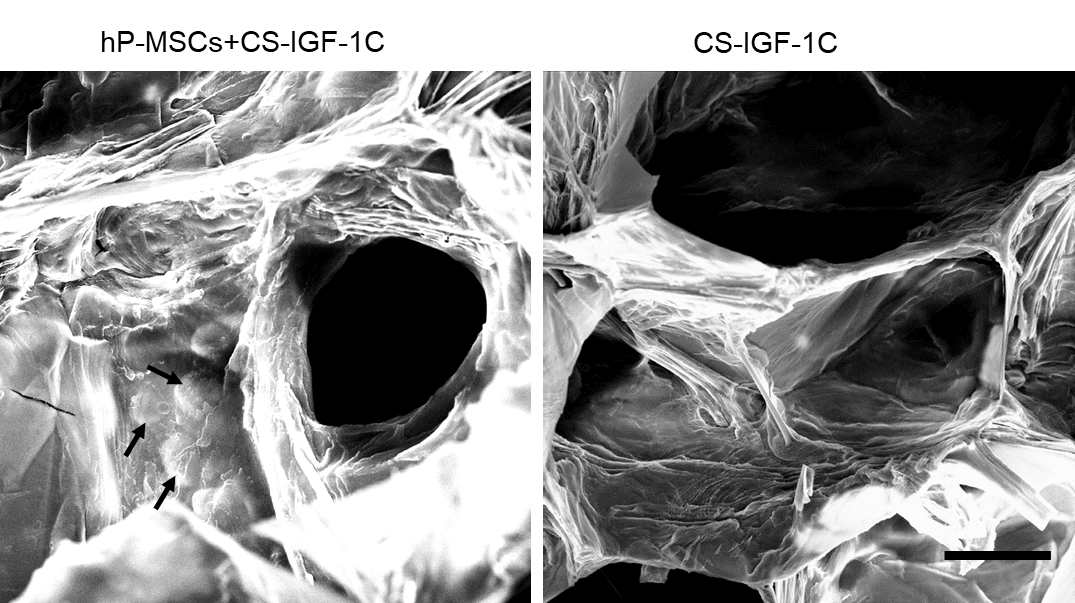
**

**Figure S2:** **Representative SEM images.** SEM images of CS-IGF-1C hydrogel with hP-MSCs (left) or not (right). Arrows indicate hP-MSCs. Scale bar, 20 μm.


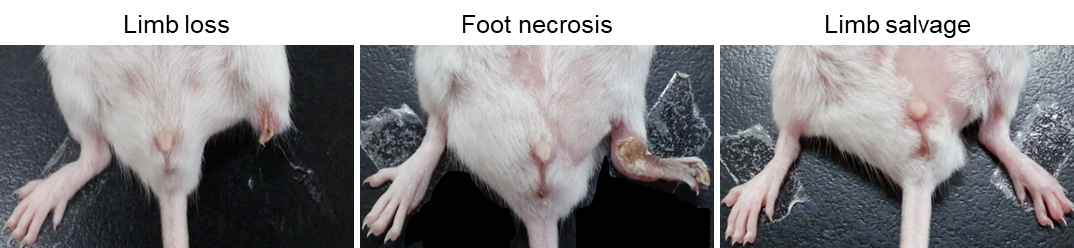


**Figure S3:** **Three outcomes** **to estimate the therapeutic effect.** The photograph of three therapeutic outcomes: limb salvage, foot necrosis, and limb loss.
